# Gut-Associated Lymphatic Tissue in Food-Restricted Rats: Influence of Refeeding and Probiotic Supplementation

**DOI:** 10.3390/microorganisms11061411

**Published:** 2023-05-26

**Authors:** Stefanie Trinh, Larissa Käver, Anna Schlösser, Anna Simon, Vanessa Kogel, Clara Voelz, Cordian Beyer, Jochen Seitz

**Affiliations:** 1 Institute of Neuroanatomy, RWTH Aachen University, Wendlingweg 2, 52074 Aachen, Germany; lkaever@ukaachen.de (L.K.); anna.schloesser@rwth-aachen.de (A.S.); anna.simon@rwth-aachen.de (A.S.); vanessa.kogel@rwth-aachen.de (V.K.); cvoelz@ukaachen.de (C.V.); cbeyer@ukaachen.de (C.B.); 2 Department of Child and Adolescent Psychiatry, Psychosomatics and Psychotherapy, RWTH Aachen University, Neuenhofer Weg 21, 52074 Aachen, Germany; jseitz@ukaachen.de

**Keywords:** activity-based anorexia, anorexia nervosa, chronic starvation, gut-associated lymphoid tissue, gut microbiome, gut permeability, Peyer plaques, probiotics

## Abstract

Anorexia nervosa (AN) is a severe and often chronic eating disorder that leads to alterations in the gut microbiome, which is known to influence several processes, such as appetite and body weight regulation, metabolism, gut permeability, inflammation, and gut–brain interactions. Using a translational activity-based anorexia (ABA) rat model, this study examined the effect of chronic food starvation, as well as multistrain probiotic supplementation and refeeding, on the structure of the gut and gut-associated lymphatic tissue (GALT). Our results indicated that ABA had an atrophic influence on intestinal morphology and increased the formation of GALT in the small bowel and colon. Higher formation of GALT in ABA rats appeared to be reversible upon application of a multistrain probiotic mixture and refeeding of the starved animals. This is the first time that increased GALT was found following starvation in the ABA model. Our results underscore a potential role of gut inflammatory alterations in the underlying pathophysiology of AN. Increased GALT could be linked to the gut microbiome, as probiotics were able to reverse this finding. These results emphasize the role of the microbiome–gut–brain axis in the pathomechanisms of AN and point to probiotics as potentially beneficial addendum in the treatment of AN.

## 1. Introduction

Anorexia nervosa (AN), a severe eating disorder, is mostly prevalent in girls and young women. It is characterized by restricted energy intake associated with extreme underweight, disturbed body perception, and anxiety about gaining weight [1]. The clinical relevance is high, as during adolescence, AN is the third most common chronic disease, with a lifetime prevalence of 1–4% in 12- to 18-year-old girls in Europe, and the mortality risk is the highest of all psychiatric disorders [1,2,3,4]. Currently, a multimodal therapeutic approach is used, including weight rehabilitation and psychotherapy, but the treatment success is limited [5]. After 7–10 years, less than 50% recover completely, while 20% of patients develop a chronic course of the disease [6,7]. Therefore, research on the underlying pathophysiology, as well as mental comorbidities, sustaining factors, and the development of new therapeutic options are urgently needed.

To study AN, the activity-based anorexia (ABA) animal model is most commonly used and well established because it mimics the main characteristics of AN, such as loss of body weight, increased physical activity, amenorrhea, and brain volume loss, by combining food starvation with running-wheel activity [8,9].

In recent years, several studies have provided evidence that periods of starvation are associated with alterations in the gut microbiome and significant shifts in its diversity in patients with AN and ABA animals [10,11,12,13,14]. In this regard, the interaction between the intestinal microbiome and the brain (“gut–brain axis”) also seems to be relevant. The latest research results indicated a causal relationship between the gut microbiome and the management of stress, anxiety disorders, and depression [15]. These results are relevant for the treatment of AN, as weight-regulatory, immunological, and psychological effects of the microbiome may influence the course and prognosis of this often chronic disease [10,16,17]. Furthermore, Jésus et al. (2014) demonstrated increased gut permeability, gut atrophy, and histological decreased thickness of the muscularis layer in ABA mice [18]. The group also found alterations in tight junction protein expressions (e.g., claudin-1, occludin, and zonulin), which are responsible for regulating intestinal permeability. Increased intestinal permeability has been reported previously in inflammatory bowel disease studied in an animal model [19] and in malnourished animals and human patients [20,21]. Corresponding to Jésus et al. (2014), Achamrah et al. (2016) also found increased colonic permeability in ABA mice [22]. Another study obtained decreased intestinal permeability in patients with AN, but the analysis method mainly assessed the small bowel permeability [23]. The intestinal barrier is responsible for filtering pathogenic microorganisms from the entire system to avoid inflammatory processes while allowing the passage of water, nutrients, and electrolytes. To maintain intestinal homeostasis and provide local immunity, gut-associated lymphoid tissue (GALT), such as aggregated Peyer’s plaques [24], are located in the intestinal tract of mammals [25,26]. GALT plays an important role in the immune response and inflammatory processes. Due to nutritional restriction related to gut microbiota dysbiosis, alterations in this mucosal immune system function could be observed in humans [27,28] and laboratory rodents [28,29].

Over the past 40 years, there has been increasing research, suggesting that probiotics can influence the immune system both in vivo and in vitro. According to the World Health Organization, probiotics are defined as “live microorganisms which when administered in adequate amounts confer a health benefit on the host” [30].

This interaction appears to be linked to the gut microbiota, as well as polysaccharide antigens and metabolites produced by these bacteria. For instance, Bifidobacteria has been found to compete with pathogenic bacteria in vitro, while a probiotic mixture comprising Lactobacilli, Bifidobacilli, and Streptococcus thermophilus has been shown to ameliorate ulcerative colitis, an inflammatory bowel disease [31,32]. Furthermore, supplementation with probiotics is known to positively influence central nervous system diseases, modify the gut microbiota, via the gut–brain axis, mediate different pathways, such as neural, hormonal, immune, inflammatory, and antioxidative signaling [33]. Probiotics are also applied in cancer therapy, since translational studies have indicated that the specific administration of selected bacterial gut species can improve the immune check-point immunotherapy clinical outcome. Lactobacillus rhamnosus GG, a commonly studied probiotic in oncology, has been found to provide benefits when given alongside anticancer treatment [34].

Based on these studies, we raised the hypothesis that administration of probiotics could potentially be beneficial as add-on treatment for AN, affecting the gut microbiome, intestinal morphology, formation of GALT, and thereby intestinal permeability [35]. Some human and animal studies have already shown the positive effects of probiotics by achieving better weight gain, maintaining appropriate mental conditions, or improving anxiety, depression, neuropsychological symptoms, and appetite levels—all relevant factors in AN [36,37,38,39,40]. Furthermore, an ongoing clinical trial examines the effects of probiotic administration in patients with AN [41]. In the present study, we analyzed the intestinal morphology, as well as the occurrence of GALT in rats, using a chronic ABA model. Furthermore, we aimed to identify whether the assumed alterations in gut permeability due to food limitations and running-wheel access could be influenced by a refeeding process or administration of probiotics.

## 2. Materials and Methods

### 2.1. Animals

**Figure 1:**
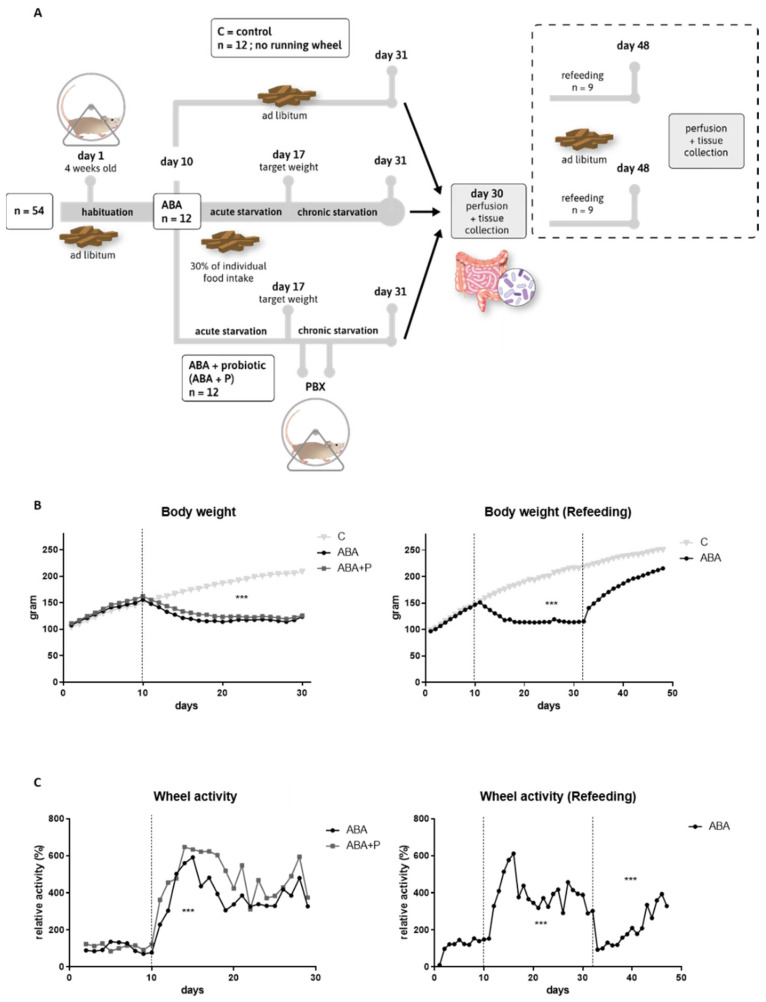
Study design, body weight, and running-wheel activity profiles. (**A**) Study design for the starvation and refeeding (dashed box) protocol. Both studies were performed with separate animals. (**B**) Mean body weight in grams per day for the starvation and refeeding study. (**C**) Mean running wheel activity normalized to the average running wheel activity of the habituation period (days 1–10) for the starvation and refeeding study. One-way ANOVA with Bonferroni correction *** = *p* ≤ 0.001. ABA = activity-based anorexia.

Four-week-old female Wistar rats (RjHan:WI, Janvier, Hannover, Germany) were commercially purchased and arrived in the animal facility with an average body weight of 103.98 g ± 8.14 g. The rats were single-housed under specific pathogen-free conditions according to DIN ISO 9001:2008 in type IV cages (1820 cm^2^, Polysulfone, Tecniplast). The cages were enriched with sawdust bedding, cotton wool pads as nesting material, and an in-cage running wheel. The room was maintained at a constant temperature (22 °C) and humidity (55%) and under a 12-h light/dark cycle (light on at 7 a.m.). During a daily handling procedure until the end of the protocol, the body weight, food intake, and running wheel activity (RWA, tachometer: Sigma BC 5.12) were recorded for each rat; 31 days delineated the chronic starvation protocol, and 48 days delineated the refeeding protocol (Figure 1A).

Thirty-six rats were used for chronic starvation experiments and randomly split into three groups: controls (C, n = 12), activity-based anorexia (ABA, n = 12), and activity-based anorexia treated with probiotics (ABA + P n = 12) (see Figure 1A). Another eighteen rats were used for refeeding experiments and divided into two groups: controls (C_ref, n = 9) and ABA with subsequent refeeding (ABA_ref, n = 9) (Figure 1A).

The animal trial was approved by the Governmental Animal Care and Use Committee (Approval no.: 84-02-04.2017.A036). All experiments were performed in accordance with the German legislation governing animal studies, following the Guide for the Care and Use of Laboratory Animals (NIH, 8th edition, 2011) and the 2010/63/EU Directive on the Protection of Animals Used for Scientific Purposes (Official Journal of the European Union, 2010).

### 2.2. Study Design

A chronic version of the ABA model was used, as previously described [42]. In brief, during the 10-day acclimatization period to habituate to single housing, and, if applicable, to the running wheel, all rats had free access to food and water. In contrast to the original ABA model developed by Routtenberg and Kuznesof [43], feeding time was not altered, but the amount of available food was reduced equivalent to 30% of the individual food intake in the habituation period for food-restricted groups (ABA, ABA + P and ABA_ref). That means a rat that had a mean food intake of 20 g per day in the habituation period, receiving 6 g per day during the acute starvation period. Control animals received food ad libitum throughout the whole experiment. Food-restricted rats received their food daily at 12 a.m. after the handling procedure. Acute food restriction was stopped when the rat lost 25% of the baseline body weight on the last day of the habituation phase (defined as the target weight), on average, after 7.13 ± 3.27 days. Subsequently, the body weight was maintained at the target weight until the end of the protocol by individually and daily adjusting the quantity of food pellets imitating chronic starvation similar to patients with AN. ABA rats had free access to the running wheel during the entire experiment. A commercially available mixture of eight different bacterial species was used as probiotic supplementation (VSL#3^®^: Bifidobacterium breve, Bifidobacterium longum, Bifidobacterium infantis, Streptococcus thermophilus, Lactobacillus acidophilus, Lactobacillus plantarum, Lactobacillus paracasei, and Lactobacillus delbrueckii subsp. bulgaricus) [44]. All rats in the ABA + P group received 1 mL (1 × 10^9^ living bacteria) on two consecutive days, as soon as the target weight was reached. For refeeding studies, ABA rats received two weeks of ad libitum access to food after the chronic starvation protocol. At the end of the respective protocol the rats were anesthetized with an overdose of isoflurane (5%, Piramal) until reflexes (tail pinch and paw pinch reflex) were no longer measurable. Body weight and running wheel activity were averaged per group and separated into the different phases of the experimental protocol (habituation: days 1–10, starvation: days 11–31, refeeding: days 32–48 (if applicable)). Both parameters were normalized to the baseline measured during the habituation period. For a graphical illustration of the study design, see Figure 1A.

### 2.3. Histological Measurements

After transcardial perfusion with artificial cerebrospinal fluid (pH = 7.4), intestinal tissue from the small intestine and the colon was extracted. Small intestine samples were taken from the duodenum, right behind the stomach. Duodenum and colon samples were dissected with scissors and washed with a 3.7% paraformaldehyde solution (pH 7.4) using a gavage syringe. For staining, the tissue specimens were rinsed with 3.7% paraformaldehyde solution (pH 7.4) for postfixation, dehydrated by an increasing alcohol/xylol series, and embedded in paraffin (Merck, Darmstadt, Germany). Finally, samples were cut on a microtome into 5 µm sections. From each animal, four sections of the duodenum and four sections of the colon were stained with hematoxylin and eosin. After deparaffinizing, sections were incubated in 0.1% hematoxylin for 4 min, followed by washing with running tap water for four minutes. Afterwards, sections were incubated in 0.2% eosin for 30 s, dehydrated in an ascending alcohol/xylol series, and mounted with DePeX (Serva, Heidelberg, Germany).

Stained slices were digitalized under a light microscope (Nikon Eclipse 80i) connected to a camera (Nikon DS-2Mv). The depth of the crypts, thickness of the lamina mucosae, and the thickness of the lamina muscularis of the colon, as well as the length of the villi and lamina muscularis of the duodenum and the surface area of GALT, were traced with the selection tool in ImageJ software (1.48v, Wayne Rasband, Kensington, MD, USA). Moreover, the incidence of GALT in each slice was counted. If a GALT plaque was present, it was additionally recorded whether the plaque reached the lumen by disrupting the lamina mucosae in the colon.

### 2.4. Gene Expression

For molecular analysis, intestinal tissue samples were snap frozen in liquid nitrogen and kept at −80 °C until further processing. After tissue collection in cryo-tubes containing 1.4 mm ceramic beads and homogenization in a FastPrep (Precelly 24, Peqlab, Erlangen, Germany) at 5000× *g* for 15 s, RNA was isolated by phenol-chloroform extraction using peqGold RNA TriFast (PeqLab, Erlangen, Germany), as previously described [45]. The purity of the obtained RNA was measured using 260/280 ratios of optical densities (Nanodrop 1000, PeqLab, Erlangen, Germany). Complementary DNA was obtained by reverse transcription using M-MLV transcriptase and random hexanucleotide primers (Invitrogen, Carlsbad, CA, USA). Relative mRNA expression levels were analyzed with quantitative real-time PCR (Bio-Rad, Feldkirchen, Germany). The ratio between the gene of interest (Occludin, sense: 5′-CCCAGGTGGCAGGTAGATTA, antisense: 5′-AGGCCTGTTTTGTGAGCACT; Claudin-1, sense: 5′-GTGGTGTTGGGTAAGAGGTTGT, antisense: 5′-AAAAGATGTGGATGGCTGTCA; Zonulin, sense: 5′-ACTGGGTCCAGGAAACAATG, antisense: 5′-TCCTCTTCCAGGGTGAATTG) and the reference gene (Cyclophilin A, sense: 5′-GGCAAATGCTGGACCAAACAC, antisense: 5′-TTAGAGTTGTCCACAGTCGGG AGATG) was calculated and expressed as the fold change relative to the control group.

### 2.5. Statistical Analysis

Differences in body weight and gut morphology (crypt depth, villi height, lamina mucosa and muscularis width, and GALT volume) between three groups (C, ABA, and ABA + P) were evaluated in a one-way analysis of variance (ANOVA) test with a Bonferroni post hoc test and Student’s *t*-test for comparison between two groups (C_ref and ABA_ref). Differences in running wheel activity before starvation and during starvation for the ABA group were also calculated by a Student’s *t*-test.

The incidence of GALT was analyzed in a linear mixed model with the assumption that missing values occurred at random, as the tissue slices could not be defined as independent. All statistical analyses were performed with SPSS version 25 (IBM, Chicago, IL, USA). The significance level was set to 5%. The results are presented as the means and standard errors of the mean (±SEM).

## 3. Results

As expected, the average body weight (*p* = 0.0001, Figure 1B) and food intake (*p* = 0.0001, Supplementary Figure S1) were significantly lower during the entire starvation period for ABA animals compared to controls. Due to the refeeding process with ad libitum food access from Day 31 onward, this effect was reversed (Figure 1B). The average body weight of the control group continuously increased throughout the experiment since the rats were still growing.

Figure 1C shows that ABA animals run significantly more during the induction of starvation than during the acclimatization period (*p* = 0.003). A similar pattern was observed during starvation in ABA animals in the refeeding group (*p* = 0.0001). Toward the end of the chronic starvation, RWA somewhat decreased, probably to prevent exhaustion, but settled on a still increased level. At the beginning of refeeding, RWA returned to baseline during the first few days, and then continuously grew again and on average increased compared to baseline (*p* = 0.0001) (Figure 1C). These results show that the ABA model was successfully applied, as important characteristics of AN, such as body weight loss, decreased food intake, and hyperactivity, were demonstrated in the studied animals.

**Figure 2:**
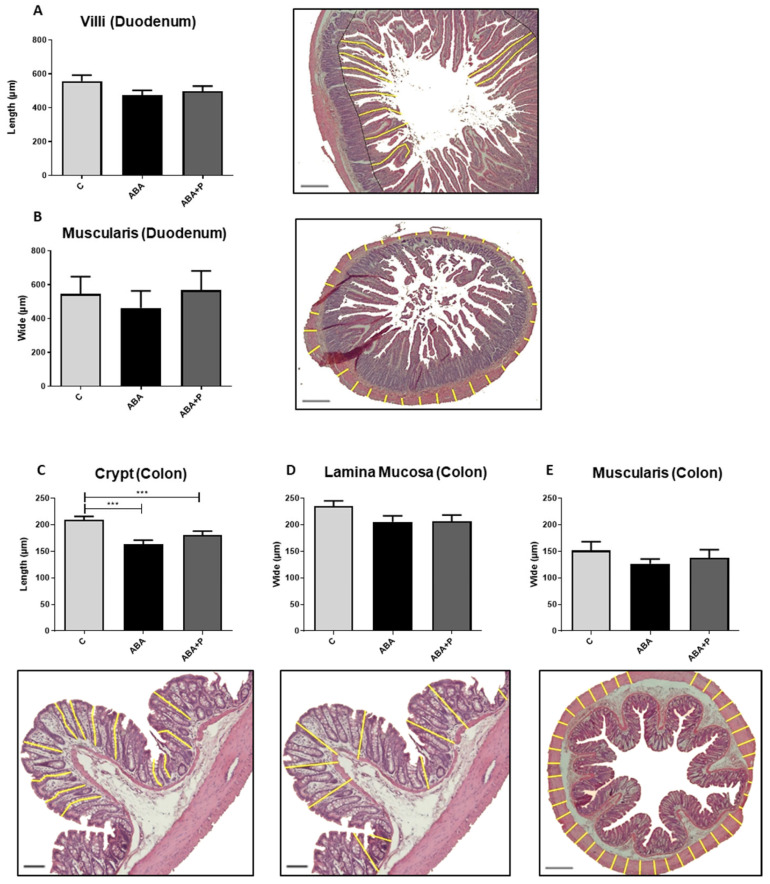
Morphology of gut tissue after starvation. (**A**) Yellow lines indicate the length of villi in the duodenum in µm and exemplary histological image with HE-staining, (**B**) yellow lines indicate the width of lamina muscularis in the duodenum in µm and exemplary histological image with HE-staining, (**C**) yellow lines indicate the crypt depth in the colon in µm and exemplary histological image with HE-staining, (**D**,**E**) yellow lines indicate the width of lamina mucosa and lamina muscularis in the colon in µm and exemplary histological images with HE-staining. One-way ANOVA with Bonferroni correction *** *p* ≤ 0.001. Scale bar = 100 µm.

All morphological parameters in both the colon and duodenum tended to be decreased in the ABA group due to starvation (Figure 2). In particular, the crypts in the colon were significantly smaller in both food-restricted groups compared to the controls (*p* = 0.0001 and *p* = 0.0012, respectively; Figure 2C). Administration of probiotics (ABA + P) led to nominal improvement in all morphological parameters when comparing the ABA + P to the ABA group. After refeeding, no differences in gut morphology could be seen in ABA rats compared to controls (Supplementary Figure S2).

**Figure 3:**
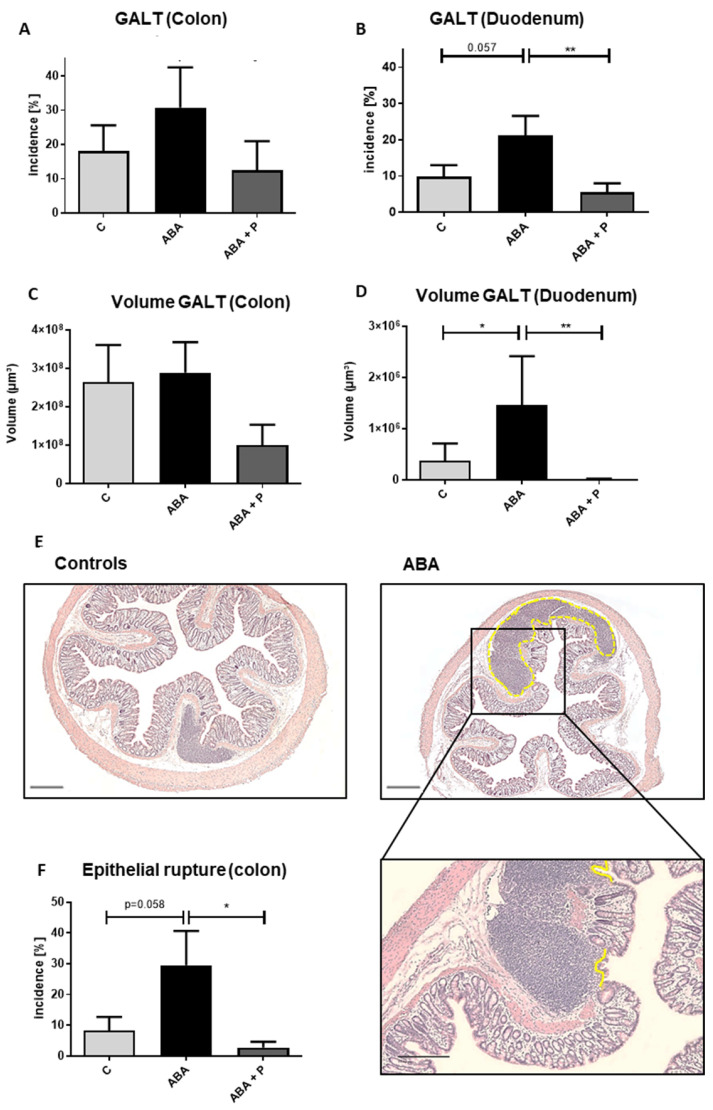
GALT after chronic starvation. Incidence in % of GALT in the (**A**) colon and (**B**) duodenum, volumes in µm^3^ of GALT in (**C**) colon and (**D**) duodenum, (**E**) histological cross section of GALT in the colon with HE-staining in control and ABA-animal, (**F**) incidence in % of epithelial rupture of Peyer plaque-like structures in the colon and exemplary histological image with HE-staining. The yellow line indicates an epithelial rupture. Linear mixed models in ANOVA * *p* ≤ 0.05, ** *p* ≤ 0.01. Scale bar = 100 µm.

In addition, we found a trend toward more lymphatic tissue in the duodenum of ABA animals compared to controls (*p* = 0.057, Figure 3B), with a similar pattern that did not reach significance in colon samples. GALT plaque volume in the duodenum was significantly enlarged in the ABA group compared to controls (Figure 3D, *p* = 0.042). Administration of probiotics led to significantly reduced GALT (incidence) in the duodenum (*p* = 0.008, Figure 3B), and a similar effect could be observed in the colon (Figure 3A). Additionally, ABA animals that received probiotic supplementation showed significantly smaller lymphatic plaques than ABA animals without probiotic supplementation in the duodenum (Figure 3D, *p* = 0.022) with a similar effect in the colon (Figure 3C). After the refeeding process, no significant differences in GALT could be detected, so the growth of aggregated lymphatic plaques seemed to be linked to starvation (Supplementary Figure S3).

Last, epithelial ruptures of lymphatic plaques through the mucosa of the colon showed a trend to be more frequent in ABA animals (*p* = 0.058). This frequency of breakthroughs was significantly lower after probiotic application in the ABA + P group (*p* = 0.016), again pointing to a potential positive effect of probiotic supplementation (Figure 3E,F).

**Figure 4:**
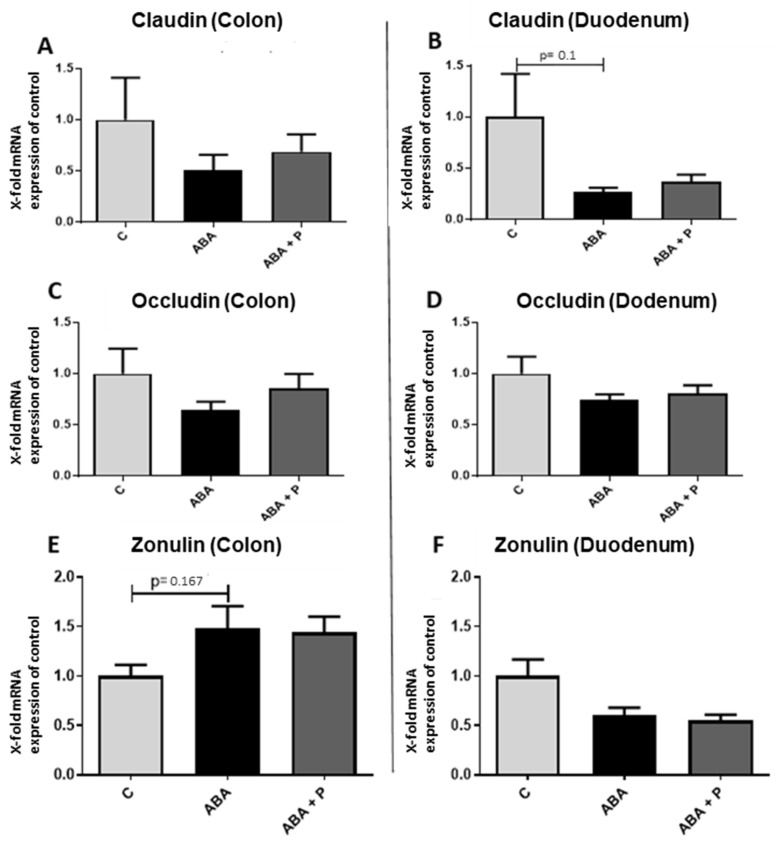
mRNA expression of the tight junction proteins occludin and claudin-1 and the regulator protein zonulin in gut tissue after starvation. mRNA expression of claudin-1 in the (**A**) colon and (**B**) duodenum, mRNA expression of occludin in (**C**) the colon and (**D**) duodenum and mRNA expression of zonulin in the (**E**) colon and (**F**) duodenum. One-way ANOVA with Bonferroni correction.

To characterize important factors influencing intestinal permeability, we determined the mRNA expression of the tight junction proteins occludin and claudin-1 [21,46], as well as the expression of the tight junction protein regulator zonulin [23] in both colon and duodenum tissue (Figure 4). The mRNA gene expression of claudin-1 and occludin tended to be reduced in ABA rats compared to controls, but did not reach statistical significance (Figure 4A–D). The zonulin expression levels appeared to show divergent results, as it tended to be more highly expressed in ABA animals and ABA + P animals than in controls in the colon, but it was less expressed in the food-restricted animals in the duodenum (Figure 4E,F).

## 4. Discussion

In the present study, we demonstrated that food restriction and running wheel activity in a translational model for AN not only lead to body weight loss and hyperactivity, but also to gut atrophy and growth of aggregated lymphatic tissue. These alterations seemed to be largely reversible after a refeeding process of chronically starved animals. Interestingly, administration of probiotics during acute starvation similarly showed a reduction in GALT, even without weight gain. These findings are in line with the hypothesis of increased gut permeability (“leaky gut”) due to atrophy caused by food restriction and running wheel activity with a consequential increase in bacterial parts or products traversing the gut wall and leading to local immune reactions. Probiotic applications associated with a reversal of gut morphological aberrations and possible reduction in immune reaction might thus be a promising route to explore for future additions to AN treatment.

### 4.1. Gut Wall Atrophy and Gut Permeability

Food starvation-induced gut atrophy and increased gut permeability were previously observed in male ABA mice by Jésus et al. (2014), suggesting that intestinal barrier dysfunction, influenced by alterations in tight junction protein expression, might be involved in the pathophysiology of AN [18]. Another study by Achamrah et al. (2016) supported these findings, as they similarly observed increased colonic permeability in ABA mice [22]. In the present study, we only observed a tendency toward a reduction in the mRNA expression of tight junction proteins, which were found to be significantly altered by Jésus et al. (2014). This could be due to methodological differences. The tight junction proteins occludin and claudin-1 regulate gut permeability [46], as well as increased intestinal permeability, due to alteration in these proteins that has been reported previously in malnourished mice and humans [20,21].

Genton et al. (2015) also determined an increased intestinal permeability due to caloric deprivation both in animals by measuring FITC-dextran fluxes, as well as in human probes by measuring their urinary recovery [28]. Interestingly, Monteleone et al. (2004) reported decreased intestinal permeability in a small study of 14 patients with AN. These results were achieved by using a lactulose/mannitol urinary test that mainly assesses small bowel permeability [23]. Gut wall atrophy and potentially increased gut permeability might facilitate the passage of pathogens into the blood circulation and, therefore, lead to an aberrant immune response also found in patients with AN and further discussed below [47,48]. This “leaky gut” could also increase the risk for developing autoimmune diseases often found in correlation with AN [49].

In our chronic starvation model, we detected gut atrophy to a higher extent in the colon than in the duodenum, which mirrors the findings of Jésus et al. (2014). This might be because of a significantly higher bacterial number and diversity in this region of the gut. Recent studies have postulated that the gut microbiome plays an important role in AN, as alterations in its diversity were found in patients with AN, as well as in ABA animals compared to controls (for a recent review, see [50]). A possible mechanism could be that increased gut permeability induced by food restriction is further aggravated by increased mucus degradation in the intestinal tract by specific microbiota, such as the genus Akkermansia muciniphila [51,52]. Higher levels of the bacterium Akkermansia were observed in patients with AN, as well as in different organisms under starvation conditions [10,51,53,54]. Mucin degradation is seen as a competitive advantage compared to other bacteria, which need dietary nutrients to survive. In addition, it might favor intestinal permeability because the protective mucus layer is disrupted.

### 4.2. Increased GALT and Its Relation to Inflammation

Potentially related to alterations in inflammatory processes and increased permeability, our results showed more and larger aggregated lymphatic tissue in ABA animals compared to controls. The assumption that RWA and food starvation have an influence on the formation of lymphatic plaques was already raised in a historic study from 1977 by Pare. Rats increased their physical activity due to food restriction, and Adan et al. (2010) postulated that this hyperactivity in acutely starved animals is associated with foraging behavior [55]. Furthermore, Paré et al. (1977) reported that starved animals had a higher probability of developing inflammatory lesions in the gastrointestinal tract (gastric ulcers) [56]. The GALT system is an important part of the peripheral mucosal immune system, as it is responsible for defending against antigenic and pathogenic material, while it recognizes commensal bacteria [26]. It is composed of highly specialized lymphoid organs (Peyer’s patches (PP) and small lymphoid aggregations) [26,57]. Aggregated lymphatic plaques are assumed to be inductive sites for intestinal immune reactions [57] and to maintain intestinal homeostasis with respect to the gut microbiome [58]. Alterations in this mucosal immune system have already been observed in response to nutritional restriction in humans [27] and laboratory rodents [29]. Nevertheless, the understanding of the development, structure, and function of GALT primarily comes from studies in animal models (reviewed in [58]), as only limited human intestinal tissue is available [59]. In a recent study, Jørgensen et al. (2021) developed a protocol to characterize distinct human GALT structures. They provided key insights into immune responses by state-of-the-art single-cell analysis, as they could determine whether such immune responses differ between the different types of GALT or within different segments of the intestine [60], which might also prove helpful in ABA and AN studies. Furthermore, a few studies have focused on inflammation, as they determined serum concentrations of cytokines in patients with AN. Meta-analyses by Dalton et al., (2020) and Solmi et al., (2015) showed that some cytokines (e.g., TNF-α, IL1-β, and IL-6) are significantly elevated, mostly in adult patients with AN [61,62]. Surprisingly, Specht et al. (2022) recently demonstrated reduced IL-1β and IL-6 serum levels and elevated TNF-α concentrations in 22 female adolescent patients with AN longitudinally at admission and discharge [63]. Regarding these results, more chronic, adult AN, but also in part adolescent AN, seems to be associated with increased pro-inflammatory cytokines. This could result from the higher gut permeability related to the higher bacterial transition and formation of GALT found in the present study. Thus, more research to understand the relationship between gut atrophy and inflammation in patients with AN is highly needed.

### 4.3. Probiotics and Refeeding Appear to Reverse Gut Atrophy and GALT

Our present research showed that administration of probiotics was associated with a significant reduction in aggregated lymphatic plaques in the small bowel in ABA animals. A systematic review by Hungin et al. (2018) proposed the use of probiotics as a therapeutic option in somatic diseases due to their beneficial effects on gastrointestinal functions [64]. In addition, probiotics have shown potential effects on the gut–brain axis, and psychological and physiological conditions, such as anxiety, depression, and appetite levels in rodents, as well as in humans, were improved [37,38,65], which are all common comorbidities of AN and, therefore, need to be considered in the treatment of this disorder. In particular, the bacterial genus Lactobacillus plantarum, which was also part of the probiotic mix used in the present study, appeared to have a potential therapeutic effect on neuropsychological disorders (e.g., depression) and gut dysbiosis, as it increased microorganisms that exert antioxidative and antidiabetic effects [66]. Regarding the significantly altered gut microbiota composition found in patients with AN [11,67,68], Liu et al. (2021) postulated in an in vitro approach that probiotics have the ability to recover the bacterial population in the intestinal tract, as well as key metabolites [37]. Weight restoration in the refeeding group had a similar effect on gut wall atrophy and GALT reduction, demonstrating their association with the starved state, and further emphasizing the critical role of weight increase in the treatment of AN.

Because weight gain, however, is often problematic and difficult to achieve in patients with AN, supplementation with probiotics might be an interesting additional option to help normalize the intestinal system.

Currently, only preliminary evidence exists on the use of probiotics in AN. Supplementation with probiotic yogurt (e.g., *L. bulgaricus*, *S. thermophilus*) has shown improvement in immune response in adolescents with AN or malnourished children [69,70,71]. Such immune modulations may also have a positive influence on the gastrointestinal tract (GI), potentially reducing GI symptoms in AN. Currently, the gut microbiome as a target for treatment is a theoretical possibility, but more research is needed.

A European consortium coordinated by our group (MiGBAN: Microbiome Gut-Brain interaction in Anorexia Nervosa) currently examines the clinical efficacy of the administration of multistrain probiotic mixture, similar to that used in this study, and omega-3 polyunsaturated fatty acids in adolescent patients with AN, hypothesizing an improvement in neuropsychological functioning in these patients compared to controls receiving placebos [41,72]. Other promising approaches to influence the gut microbiome, potentially GALT and gut permeability, are supplementation with short-chain fatty acids (SCFAs), as they are supposed to have anti-inflammatory effects on the gut [73,74,75] as well as fecal microbiota transplantation. There are two case reports using fecal microbiota transplantation in patients with AN, showing weight gain in one and an improved gut barrier, as well as increased diversity of the microbiome, but without clinical improvement in the other case [76,77].

### 4.4. Limitations

The ABA animal model is a well established experimental model that mimics psychological and physiological characteristics of AN and was successfully applied in the present study [42]. Nevertheless, caution is required when translating animal research to human patients, among others, because cognitive components or personal characteristics (e.g., perfectionism, control compulsion, or body perception disorder) found in patients with AN cannot be evoked in any animal study design. However, the translational importance is estimated to be high as a multitude of symptoms similar to AN can be evoked, and it is the most commonly used animal model to study AN [78,79]. In addition, this model seems purposeful to study intestinal morphology and GALT as well. In future studies, it would be interesting to isolate lymphatic patches from the gut tissue and to study the exact cell composition.

## 5. Conclusions

Food restriction and increased locomotor activity had an influence on gut atrophy and the formation of GALT in this animal study, which fits well with previous research data and might help to explain potentially increased gut permeability. For the first time, we showed increased formation of GALT in the ABA model, which could be induced by more bacterial parts and products traversing the gut wall. These lymphatic plaques might also play a role in the dysregulated balance of pro- and anti-inflammatory cytokines found in patients with AN. Furthermore, our refeeding study supports the importance of weight restoration as a key factor in the treatment of AN, as morphological parameters and growth of aggregated lymphatic plaques seem to be reversible due to refeeding. The observed alterations in the lymphatic plaques were further improved by administration of multistrain probiotics. These results help to explain important parts of the underlying pathophysiology regarding gut permeability and inflammation in the ABA model and potentially AN, and they might help to develop microbiome-targeted interventions, such as probiotics, to be added to AN treatment.

## Data Availability

The data presented in this study are available upon request from the corresponding author.

## Supplementary Materials

The following supporting information can be downloaded at: https://www.mdpi.com/article/10.3390/microorganisms11061411/s1, Figure S1: Mean food intake in grams per day for the starvation and refeeding study; Figure S2: Morphology of gut tissue after refeeding, Figure S3: GALT after refeeding.
