# Supplementary material for: Gut-Associated Lymphatic Tissue in Food-Restricted Rats: Influence of Refeeding and Probiotic Supplementation
Source: Microorganisms. 2023 May 26;11(6):1411. doi: 10.3390/microorganisms11061411 (PMC10304055; doi:10.3390/microorganisms11061411)
Supplement: Supplementary file 1 [file microorganisms-11-01411-s001.zip › microorganisms-2329512-supplementary.pdf]

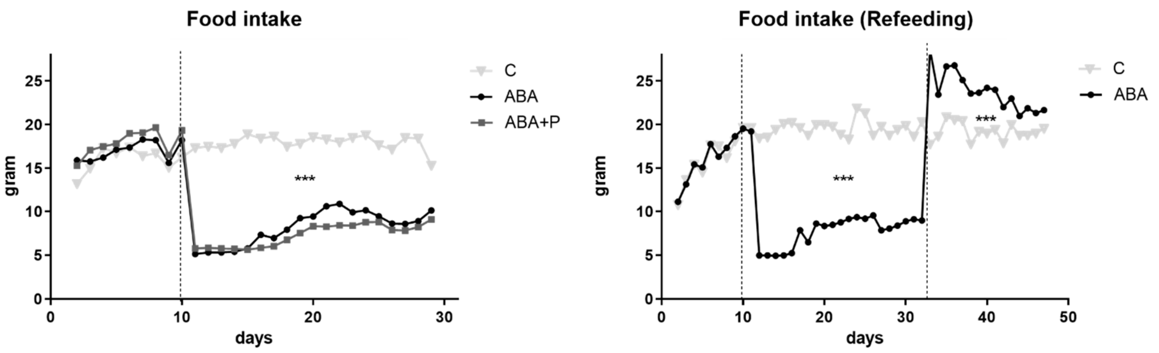

Figure S1. Mean food intake in grams per day for the starvation and refeeding study. One-way ANOVA with Bonferroni correction *** $p \leq 0.001$.

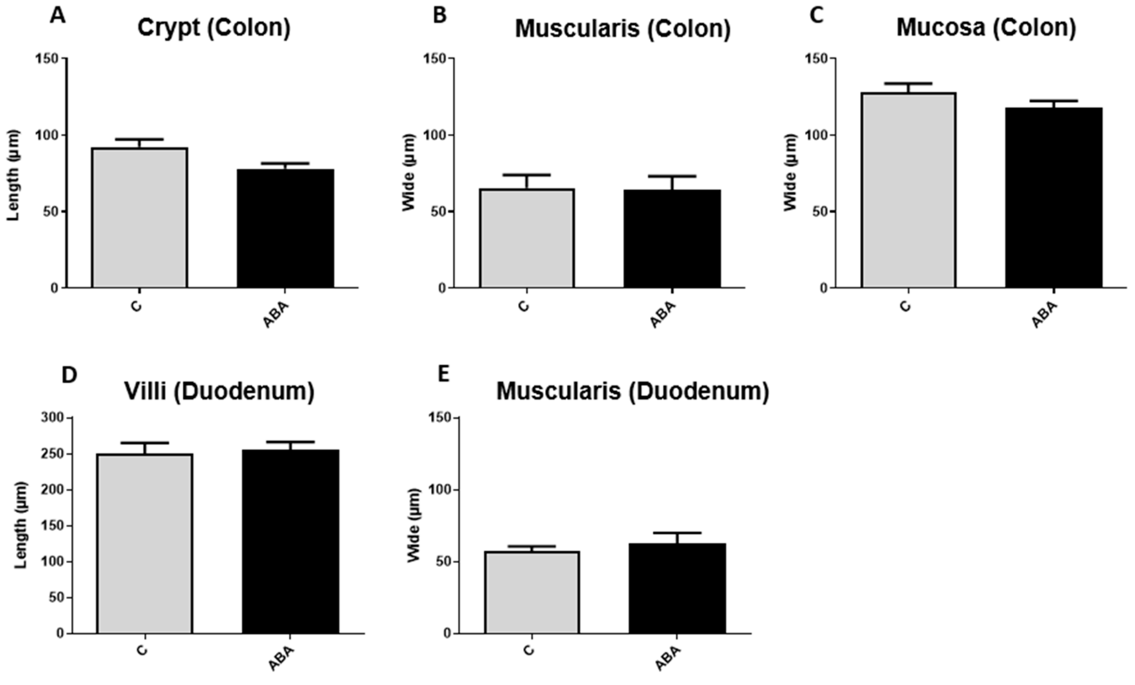

Figure S2. Morphology of gut tissue after refeeding.

(A) crypt depth in the colon in µm, (B + C) width of lamina mucosa and lamina muscularis in the colon in µm, (D) length of villi in the duodenum in µm and (B) width of lamina muscularis in the duodenum in µm. Student´s t-test.

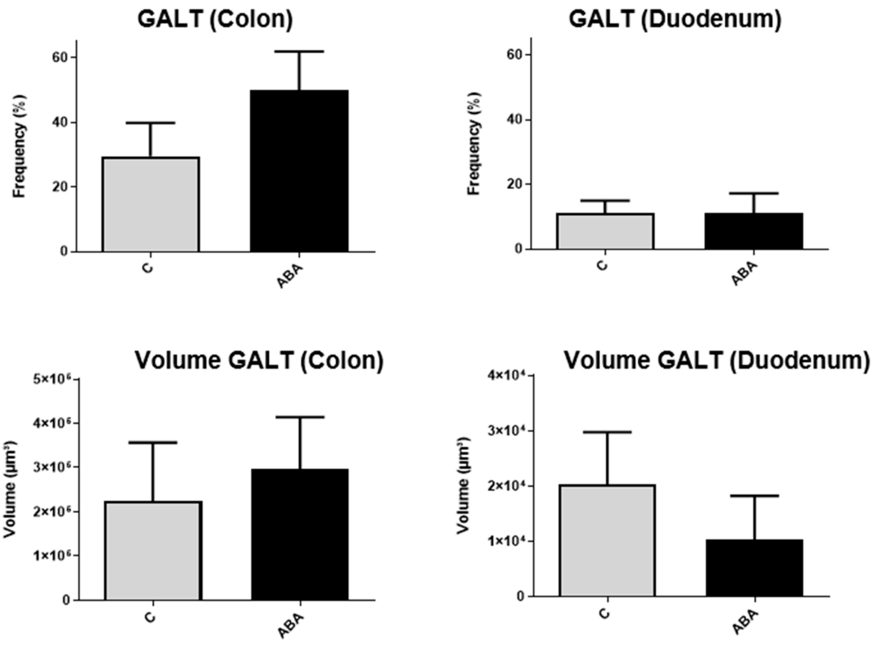

Figure S3. GALT after refeeding.

Incidence in % of GALT in the (A) colon and (B) duodenum; volumess in μm³ of GALT in (C) colon and (D) duodenum. Student´s t-test.
